# Large *Shigella flexneri* outbreak linked to a takeaway, South Wales: a case–control study

**DOI:** 10.1017/S0950268824000943

**Published:** 2025-02-14

**Authors:** Amy Plimmer, Laia Fina, Oghogho Orife, Beverley Griggs, Maria Saavedra-Campos, Donall Forde, Cerys Edwards, Louise Driscoll, Ananda Giri Shankar, Daniel Thomas

**Affiliations:** 1 UK Health Security Agency, London; 2 Health Security Agency, UK; 3 Public Health Wales (PHW), Communicable Disease Surveillance Centre, Cardiff, UK; 4 Public Health Wales (PHW), Health Protection Team, Cardiff, UK; 5 Aneurin Bevan University Health Board Microbiology, NHS, Newport, UK; 6 Monmouthshire County Council, UK

**Keywords:** community outbreaks, epidemiology, Shigella, case–control

## Abstract

In February 2023, 52 cases of gastrointestinal illness were reported in customers of Takeaway A, South Wales. *Shigella flexneri* serotype 2a was the causative organism. An outbreak investigation was conducted to determine the extent and vehicle of the outbreak.

Following descriptive summary and environmental investigations, a case–control study was completed. Participants completed a telephone questionnaire on food, travel, and environmental exposures. A multivariable logistic regression model was built, including exposures with *p*-values < 0.2 and interactions identified on stratified analysis. Staff faecal samples were screened for *Shigella* sp.

Thirty-one cases and 29 controls were included in the study. Eighty-seven per cent of cases and 76% of controls ate from Takeaway A on 10 February 2023. Coleslaw was the main factor associated with illness (aOR: 200, 95% CI: 12–3220) and an interaction with cabbage was identified (aOR: 886, 95% CI: 26–30034). *Shigella* sp. were not detected in any staff samples.

Coleslaw was the most likely vehicle. Though the contamination route is unknown, a food handler is the most likely source. This large outbreak differs from recent European outbreaks, which primarily have been associated with sexual transmission. Although uncommon in the UK, *S. flexneri* should be considered as a cause of foodborne outbreaks.

## Introduction

Shigella is a gram-negative bacterium responsible for the gastrointestinal illness, shigellosis [1]. The annual incidence of Shigella infection is estimated at 30 per 100000 population globally. Of the four species of Shigella, *Shigella sonnei* is most common in Europe, followed by *Shigella flexneri.* Both are characterized by ‘bacillary dysentery’, including profuse, sometimes bloody diarrhoea, fever, and vomiting [2]. *S. flexneri* is often more severe than *S. sonnei.* Approximately 2% of people infected with *S. flexneri* go on to develop post-infectious arthritis (Reiter’s syndrome) [3].

In 2022, a rise in *Shigella* sp. laboratory reports was recorded in Wales. This trend was matched by an increase in *S. flexneri* observed in England in 2021–2022, as reported by the United Kingdom Health Security Agency (UKHSA) [4]. Nearly 70% of *S. flexneri* cases reported to UKHSA in the first half of 2022 were in men who have sex with men (MSM) [4]. Where outbreaks of *S. flexneri* in the UK have been published, more often than not, these outbreaks focus on transmission via sexual contact between MSM [5, 6].

### The alert

On 17 February 2023, Public Health Wales (PHW) Health Protection Team (HPT) was contacted by the microbiology laboratory in Aneurin Bevan University Health Board, following the identification of nine new reports of presumptive Shigella by PCR test on faecal samples. The cases, who had experienced gastrointestinal symptoms including diarrhoea and vomiting, were resident in two neighbouring local authorities in South-East Wales. Four of the nine faecal samples were requested by the same general practice (GP) surgery. Four cases had been hospitalized as a result of their symptoms.

Environmental Health Officers (EHOs) undertook risk assessments of cases and completed standardized exposure questionnaires. From this initial investigation, all nine cases reported a common link. All had eaten food from the same takeaway (Takeaway A) on the 10 or 11 February 2023, with symptom onsets reported between 12 and 13 February 2023. An outbreak control team (OCT) was convened, where microbiology colleagues reported that two samples had undergone additional culture testing, identifying the causative organism, *S. flexneri* serotype 2a.

To further understand the exposures associated with illness, PHW Communicable Disease Surveillance Centre (CDSC) conducted a case–control study to investigate this outbreak. The aim of this investigation was to determine the source of the *S. flexneri* outbreak, including any food vehicle, in order to inform the OCT and support management of this and future outbreaks of this nature/setting.

## Methods

### Case definition

We defined cases as possible, probable, or confirmed using the definitions shown in Table 1. Cases were further defined in terms of their exposure to food from Takeaway A (Table 1). Cases with direct exposure to food from Takeaway A on 10–11 February were defined as primary exposure, whereas cases who had not consumed food from the takeaway on the dates of concern were defined as secondary exposure.

**Table 1.** Case and exposure definitions for shigellosis outbreak linked to takeaway in South Wales, February 2023

Case	Definition
Possible	A person with GI illness compatible with bacillary dysentery, with symptom onset from 10 February 2023, *AND* Resident of Town A or Town B in South-East Wales or surrounding areas
Probable	*As above, plus:* An epidemiological link to the incident, including: Contact of a known case *OR* Ate food purchased from Takeaway A between 10 and 11 February 2023, *OR* A Shigella PCR (ipaH) positive result from 10 February 2023
Confirmed	*As above, plus:* A culture positive result for *Shigella flexneri*
**Exposure**	**Definition**
Primary	Cases fitting any possible, probable or confirmed definitions above AND had consumed food from Takeaway A on 10–11 February 2023
Secondary	Cases fitting any possible, probable or confirmed definitions above AND ***Did not*** consume food from Takeaway A on 10–11 February 2023

### Descriptive epidemiology

A line list was maintained, including demographic, epidemiological, and microbiological information on all cases. Data were cleaned and analysed using Stata 14.2 [7] and R [8]. Cases were described in terms of person, place and time, and an epidemiological curve was constructed.

### Whole genome sequencing

Samples from 28 confirmed *S. flexneri* cases were sent to UKHSA Gastro-Bacterial Reference Unit (GBRU) for whole genome sequencing (WGS). The resultant single nucleotide polymorphism (SNP) addresses were returned to PHW on 7 March 2023. A threshold of 10-SNPs difference across the core-genome sequences is used to define likely transmission clusters in routine public health surveillance of Shigella in England and Wales [9].

### Environmental investigations

EHOs visited Takeaway A on 17 February 2023 and conducted an inspection. As part of the inspection, all five staff from Takeaway A were asked to provide stool samples, which were tested for the presence of Shigella by PCR. In addition, six household contacts of Takeaway A staff were also asked to provide stool samples to exclude possible transmission between the home environment and the takeaway.

## Analytical study

### Study design and hypothesis

We conducted a case–control study. The alternative hypothesis (H_1_) of this analytical study was that shigellosis was associated with the consumption of a food item from Takeaway A between 10 and 11 February 2023.

The null hypothesis (H_0_) was that shigellosis was not associated with the consumption of food from Takeaway A.

This hypothesis was tested using an unmatched case–control study design.

### Recruitment of cases and controls

Probable or confirmed cases with laboratory confirmation of *Shigella* sp. or *S. flexneri* were eligible for inclusion in the case–control study. Cases without laboratory confirmation of a Shigella infection were excluded, as were cases with evidence of secondary exposure, as defined in Table 1. One confirmed case resident outside Wales was excluded from the analytical study.

Controls were defined and selected as per the definitions in Table 2. Controls were identified during case recruitment using traditional snowballing methods (i.e., asymptomatic friends and family of cases with primary exposure to Takeaway A). These were supplemented with controls unrelated to known cases, who had ordered food from Takeaway A via an online application (app) on 10–11 February 2023. Account name and telephone numbers of app users who had ordered from the takeaway were requested by PHW-HPT, citing statutory responsibilities of PHW to gather this information for outbreak investigations. The app owner shared these data with the OCT on 13 March 2023.

**Table 2.** Control definition and selection methods for shigellosis outbreak linked to takeaway in South Wales, February 2023

Term	Definition
Control	A person who had eaten food from Takeaway A between 10 and 11 February 2023 *AND* Did not develop gastrointestinal symptoms *AND* Did not test positive for Shigella on PCR.
	**Selection methods:** Family or friends of possible, probable or confirmed cases Customers who had ordered food from Takeaway A via an online application (app) on 10–11 February 2023 Persons who had eaten in the same party as controls who had ordered food via the takeaways’ app on 10–11 February 2023, as identified by app users.

Where control recruitment led to the identification of symptomatic persons, these were categorized as cases as per definitions in Table 1. If these persons met the definition of confirmed or probable cases, which included laboratory confirmation of a Shigella infection, they were included as cases in the case–control study.

Further explanation of the recruitment process for the analytical study is included in Supplementary Data (S1).

### Questionnaire development

To test the hypothesis that illness was associated with consumption of a food item at Takeaway A and support the identification of exposures associated with illness, a questionnaire was developed to collect information from cases and controls on food items eaten from Takeaway A, including portion size, as well as symptom presentation and severity. A menu of food items available for order was obtained from Takeaway A’s webpage, and ingredient lists for sauces and condiments were obtained by EHOs during discussions with the takeaway. To explore other potential common exposures for the cases other than Takeaway A, the questionnaire also asked about visits to other food establishments (restaurants/takeaways/cafes), grocery shopping, occupation, and travel history. Questionnaires were delivered over the telephone by trained CDSC team members. Interviews were conducted between 1 and 20 March 2023. Responses were uploaded to the appropriate case or control record on the PHW case incident management system.

### Analytical epidemiology

Questionnaire data were cleaned in R [8] and Stata 14.2 [7]. Response rates of cases and controls were calculated. Chi-squared tests (*t*-tests for age) were calculated to estimate the differences between case and control respondents versus nonrespondents. Included cases and controls were described in terms of age, sex, postcode area of residence, and date of order from Takeaway A (i.e., exposure date). Chi-squared tests (*t*-tests for age) were used to compare cases and controls and to inform later analytical calculations.

Univariable analysis was conducted on the exposures listed in the questionnaire. Percentage of cases and controls exposed to each item were calculated. Odds ratios (ORs) and 95% confidence intervals (95%CI) with *p*-values were calculated for each item to identify statistically significant associations (*p* < 0.05).

Items from the univariable analysis with a *p*-value < 0.2 were investigated further by stratification to identify potential confounders and effect modifiers. The exposures tested in the stratified analysis were added into a multivariable logistic regression model in a forward-stepwise approach to identify the most likely vehicles of transmission and calculate adjusted OR (aOR). Likelihood ratio tests were calculated to assess the fit of the models as new variables were added to them.

### Additional analysis

A dose–response analysis was conducted on variables included in the multivariable model to identify if consumption of larger portions increased the odds of illness. OR with 95%CI was calculated to identify statistically significant associations (*p* < 0.05) between cases and controls exposed to variables from the multivariable model, and a test for homogeneity (Chi-squared test) was completed. Additionally, these variables were also included in a dose severity analysis to ascertain whether greater exposure contributed to the onset of bloody diarrhoea or cases requiring hospitalization. OR with 95% CI were calculated for cases with and without bloody diarrhoea per dose exposed and for cases hospitalized versus not hospitalized.

To investigate whether there was gender-specific recall of consumption, we conducted a gender-response analysis between variables included in the final multivariable model to ascertain whether gender modified odds of exposure to another variable in the model. Chi-squared tests identified any significant association between the variable under test and gender.

## Ethics and information governance

As this work was performed as part of statutory service delivery duty in response to an outbreak of communicable disease, no research ethics approval was required. All documents and files containing person-identifiable information were handled and stored in compliance with the Data Protection Act (1998) and GDPR (2018) as well as by guidelines established by the local Caldicott guardian.

## Results

### Descriptive findings

In total, 52 cases were linked to the outbreak (Table 3). Twenty-nine (56%) met the definition for a confirmed case with a culture-positive result for *S. flexneri.* Of 23 probable cases (44%), 8 (35%) had a PCR sample positive for *Shigella* sp. Females accounted for 30 cases (58%). Median case age was 41 years (range: 1–75 years, Interquartile range: 16–63 years). Eleven cases (21%) were hospitalized as a result of their symptoms. All except one case lived in South-East Wales. The remaining case lived in South-West England.

**Table 3.** Summary of cases linked to outbreak (*n* = 52)

Exposure	*n*	%
Female	30	58
Confirmed^a^	29	56
Probable	23	44
PCR positive	8	35
PCR negative^b^	2	9
No sample	13	57
Hospitalised	11	21
Ate at Takeaway A	50	96
Exposure date		
10 February 2023	40	77
11 February 2023	10	19
Residential location^c^		
Town A	37	71
Town B	10	19
Town C	3	6
Town D	1	2
Outside Wales	1	2
Age/years (mean, range)	41 (1–75)	

a 1 confirmed case was secondary transmission, had not eaten at Takeaway A.

b 1 probable PCR negative case was secondary transmission, had not eaten at Takeaway A.

c All towns are in South-East Wales, unless otherwise specified.

**Figure 1.**
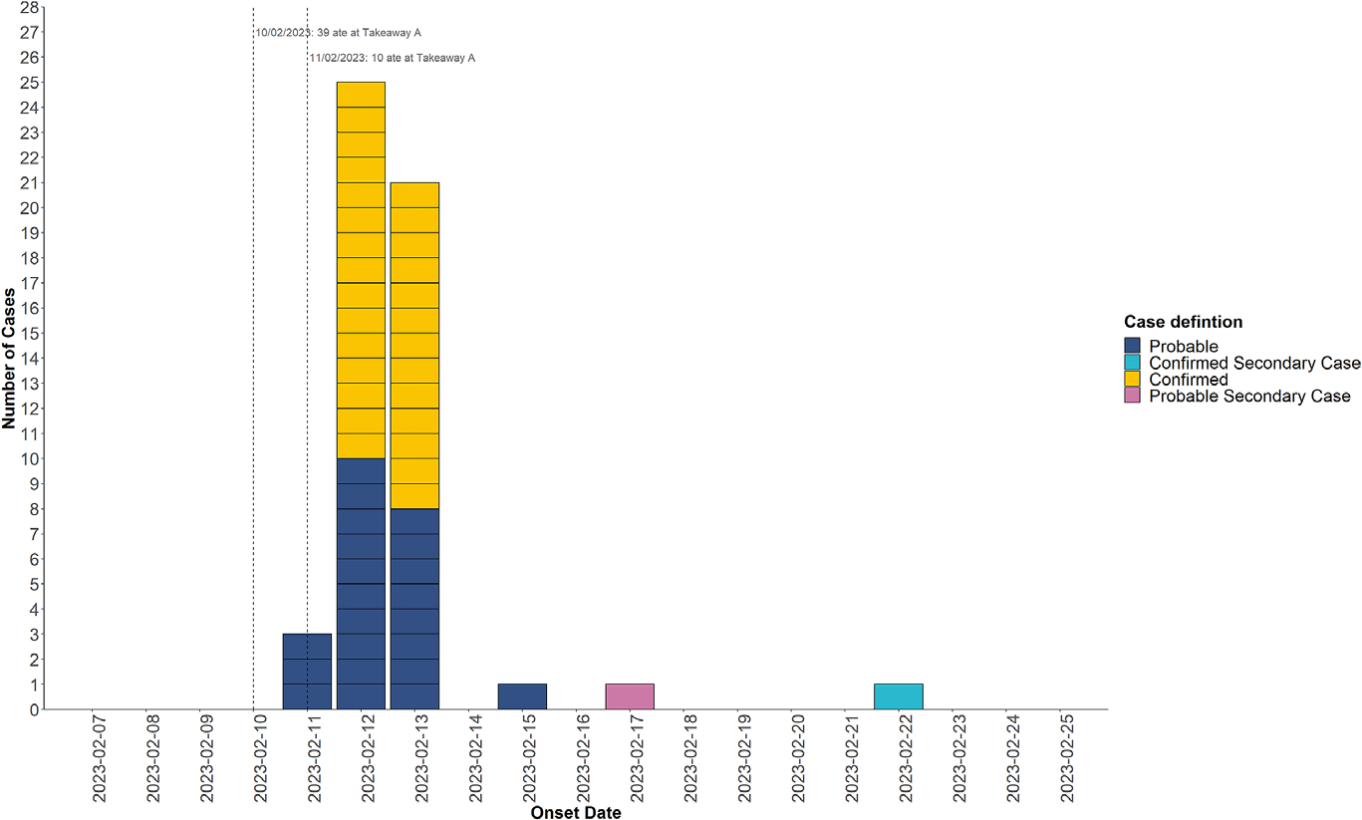
Epidemiological curve of confirmed and probable cases linked to the outbreak.

Cases symptom onsets were tightly clustered, with symptoms starting within 24–48 hours after exposure to Takeaway A (Figure 1). Of 52 cases, 50 (96%) had eaten at the Takeaway A, the majority of which (*n* = 40, 77%) ate food from the premises on the 10 February 2023. Two secondary exposure cases, including one culture-positive case and one probable PCR-negative case, did not eat at Takeaway A. These cases were thought to have been exposed to *Shigella* sp. via direct contact with primary exposure cases of people who had eaten from the takeaway. Cases with secondary exposure had symptom onset later in the outbreak, between 2 and 7 days after all cases with primary exposure reported symptom onset.

### Whole genome sequencing

Sequenced case samples were clustered within five SNPs. Comparison to available genomic data on *S. flexneri* showed that this particular genomic profile had not been reported elsewhere in England or Wales—either within the time frame of the investigation, or since the establishment of routine WGS for Shigella isolates in the UK in 2015.

### Environmental investigations

The EHOs’ inspection of Takeaway A identified missed opportunities for hand washing and equipment cleaning between handling raw and ready-to-eat foods, including dirty and washed vegetables. Temperature control in the display fridge exceeded UK standards of 8°C [10]. This was previously noted during a routine inspection of the premises in May 2022. The business voluntarily closed from 12 to 16 February 2023 for planned refurbishment and cleaning, so it was not possible to sample food preparation services. It was also not possible for EHOs to collect food samples for testing; however, it was noted that salad items were prepared fresh onsite daily, including coleslaw (Supplementary Figure S2). Different cabbage types were used for salad cabbage and the coleslaw—red cabbage for salad, white cabbage for coleslaw. On this occasion, both cabbage types were shredded mechanically. Once prepared, these items were kept in the display chiller.

All five staff provided stool samples on two separate occasions after the EHO inspection. These were all negative for Shigella. Stool samples provided by six close contacts of staff were also negative for Shigella.

### Case–control study

Of cases described above, 34 were eligible for inclusion in the case–control study (27 confirmed cases, 7 probable cases). Of eligible cases, 31 completed the questionnaire, whilst three cases were not contactable. This gives a response rate of 91%. Of the 44 eligible controls identified by cases (*n* = 18) and app data/parties of app users (*n* = 26), 29 controls successfully completed telephone questionnaires, with a response rate of 66% (Supplementary Figure S1). No significant difference was identified between the age, sex, and residential location of respondent and non-respondent cases, and there was no significant difference between the age and sex of respondent and non-respondent controls.

Cases participating in the analytical study were significantly older than controls (mean age: cases: 43 years, range 11–75 years; controls: 33 years, range 12–67 years; *p* = 0.024). Participating cases also included significantly fewer men than controls (male cases: 10, 32%; male controls: 18, 62%, *p* = 0.021). Both factors were included in the analytical analysis. There was no significant difference in the location of residence or date of exposure to Takeaway A between cases and controls.

### Univariable analysis

The results of univariable analysis of tested exposures, where the calculated OR exceed 1.0 and *p* < 0.2, are shown in Table 4. Of menu options from Takeaway A, 5/35 had an elevated OR significantly associated (*p* < 0.05) with being a case. In particular, coleslaw—which was eaten by 27 cases (87%) and 8 controls (28%)—had an OR of 17.7 (95% CI 4–87, *p* < 0.0001). Univariable analysis also identified that being female was significantly associated with illness. The odds of being a case were 3.4 times greater in females compared to males (95% CI 1–11, *p* < 0.021). No other significant exposures were associated with the outcome, including other food eaten outside the home, grocery shopping locations, and travel.

**Table 4.** Exposures of cases (*n* = 31) and controls (*n* = 29) in the case–control study with an odds ratio (OR) < 1 and a *p*-value < 0.2

Exposure	Cases		Controls		OR	95% CI	*p*-value
	*n*	%	*n*	%			
**Takeaway menu**							
Coleslaw	27	87.1	8	27.6	17.8	4.09–87.22	0.000
Garlic Mayonnaise	27	87.1	17	58.6	4.8	1.16–23.04	0.013
Doner Kebab	8	25.8	2	6.9	4.7	0.80–48.55	0.050
Cabbage	18	58.1	7	24.1	4.4	1.27–15.55	0.008
Lettuce	26	83.9	16	55.2	4.2	1.12–17.71	0.015
Cucumber	24	77.4	14	48.3	3.7	1.07–13.17	0.019
Onion	20	64.5	12	41.4	2.6	0.81–8.33	0.073
**Other**							
Female	21	67.7	11	37.9	3.4	1.05–11.41	0.021

### Stratified analysis

To assess for effect modifiers and/or confounders, exposures with calculated p-values<0.2 in the univariable analysis were stratified by exposure to coleslaw. The results of this stratified analysis are in Table 5. The presence of a significant result to the test of homogeneity (i.e. *p* < 0.05) for cabbage (*p* = 0.02) and cucumber (*p* = 0.01) suggests the effect of coleslaw was being modified by these items. Likewise, the presence of an association between illness and garlic mayonnaise in the absence of coleslaw (lower 95%CI in unexposed strata = 1.17) suggests effect modification. Interaction between these food items and coleslaw were considered when building the multivariable model.

**Table 5.** Stratification of key exposures, by exposure to coleslaw

Exposure	Stratum-specific OR (95%CI)		Test for homogeneity	Crude OR (95% CI)	Adjusted OR (95% CI)	Change (%)
	Exposed	Unexposed				
Garlic Mayonnaise	0.0 (0.0–3.2)	(1.2–)	–	4.8 (1.2–23.0)	2.1 (0.4–11.1)	−55.9
Cabbage	0.8 (0.1–4.9)	28.5 (1.2–1569.5)	0.02	4.4 (1.3–15.6)	2.0 (0.6–6.7)	−54.3
Lettuce	0.0 (0.0–3.2)	4.9 (0.3–275.1)	–	4.2 (1.1–17.7)	1.3 (0.2–6.5)	−70.1
Cucumber	0.5 (0.0–5.5)	6.0 (0.4–336.9)	0.01	3.7 (1.1–13.2)	1.5 (0.4–6.3)	−58.1
Onion	0.6 (0.1–4.1)	7.5 (0.5–419.2)	0.09	2.6 (0.8–8.3)	1.4 (0.4–5.1)	−44.9
Doner Kebab	1.1 (0.1–13.0)	(0.0–)	–	4.7 (0.8–48.6)	1.8 (0.4–9.1)	−62.0
Female	6.0 (0.8–68.6)	4.0 (0.3–227.1)	0.79	3.4 (1.1–11.4)	5.2 (1.2–21.9)	50.6

### Multivariable analysis

The final multivariable logistic regression model is in Table 6. Two menu items were identified as independent risk factors for infection: coleslaw (aOR 200, 95% CI 12–3220, *p* < 0.001) and cabbage (aOR 71, 95% CI 3–1580, *p* = 0.007). When both coleslaw and cabbage were included as an interaction term, the adjusted odds of being a case were substantially greater (aOR 886, 95% CI 26–30034, *p* < 0.001). The model also included gender as an independent factor. Being female was associated with a higher odds of illness, when adjusting for all other variables (aOR 10, 95% CI 2–58, *p* = 0.01). No other menu items improved the fit of the model.

**Table 6.** Multivariable logistic regression model

Exposure	Adjusted OR	95% CI	*p*-value
Coleslaw only	199.9	12.4–3219.9	<0.001
Cabbage only	71.2	3.2–1579.6	0.007
Coleslaw and cabbage	886.2	26.1–30033.6	<0.001
Female	9.9	1.7–57.7	0.011

### Dose–response effect of coleslaw consumption

Coleslaw portion sizes ordered by cases and controls were compared to identify any dose–response effect. The test for homogeneity showed there was a significant difference between the odds of illness in people who ate only a small portion of coleslaw compared to those who ate no coleslaw (Chi^2^: 21.75, *p* < 0.0001). Odds of illness were elevated in those who ordered large portions of coleslaw, but not significantly and not to the same extent as those who ate small portions.

### Dose-severity analysis of coleslaw consumption

A dose-severity analysis compared coleslaw consumption and reports of bloody diarrhoea amongst cases included in the analytical study (*n* = 34, 75%). The analysis showed a significant association between those who ate coleslaw and those who reported bloody diarrhoea (OR 6.23, 95% CI 1.6–29, *p* = 0.003). Odds of illness were elevated in those who ordered large portions of coleslaw but not significantly (OR 3.5, 95% CI 0.6–18, *p* = 0.14). A significant association was observed amongst those who ate small portions of coleslaw (OR 7.9, 95% CI 2–30, *p* = 0.002).

A second dose-severity analysis compared coleslaw consumption and hospitalization of cases (*n* = 9). The odds of exposure to coleslaw amongst hospitalized cases were seven times greater than those of cases or controls who were not hospitalized. However, despite a significant p-value, this association should be interpreted with caution as the confidence intervals cross 1 (95% CI 0.83–328, *p* = 0.04). The odds of hospitalization increased with portion size; however, this relationship was not significant (Small: OR 6, 95% CI 0.7–56, *p* = 0.115; large: OR 10.3, 95% CI 0.9–155, *p* = 0.06).

### Gender bias of coleslaw orders

The multivariable model identified being female as an independent risk factor associated with illness. We tested if the reporting of coleslaw orders was affected by a gender bias. This calculation was not significant (Chi^2^: 0.49, *p* = 0.48). The test was repeated with cabbage orders, which was also not significant (Chi^2^: 0.76, *p* = 0.38).

## Discussion

In the UK, outbreaks of *S. flexneri* with foodborne transmissions are rare [11]. Here, we describe a large outbreak linked to the consumption of food from a takeaway.

The close symptom onset of primary cases suggests a point source. The epidemic curve may have been truncated by the pre-planned closure of Takeaway A for refurbishment. However, the lack of cases following this closure could also indicate that the exposure likely occurred in Takeaway A in the period immediately before the closure. A point source outbreak is supported by a high level of relatedness amongst samples that underwent WGS, suggesting a common exposure led to transmission. Shigella isolates are considered to be linked to a common exposure, place or event, at the 10-SNP level [9]. The isolates in this investigation were related at the 5-SNP level, and a low amount of genetic diversity was identified. UKHSA-GBRU reported no genomic similarities between this cluster and clusters of *S. flexneri* circulating in Wales or England, or *S. flexneri* clusters associated with travel to a specific region, or with the MSM community.

Secondly, the case–control study identified coleslaw from Takeaway A as the most likely vehicle for infection. The high-fat content and large surface area of coleslaw make it an ideal vector for the transmission of foodborne illness [12]. Coleslaw has previously been recognized as the likely source of illness in other foodborne outbreaks, including norovirus and listeriosis [13, 14]. It is well documented that *Shigella* sp. can survive for approximately 5–10 days on acidic food [15], with the potential to survive on inanimate surfaces for up to 5 months [16]. However, humans are the only significant reservoir of Shigella infection [2]. It is unclear how the coleslaw became contaminated in this outbreak. Inspection of the takeaway by EHOs noted that the coleslaw was made fresh on-site daily, as opposed to the takeaway purchasing a ready-to-eat product. Environmental samples from food preparation surfaces were not collected in this outbreak. As part of the outbreak investigation, all staff members and six families of staff were potted, but microbiological confirmation of Shigella infection was not identified in any of these samples. However, as Shigella has a very low infective dose – 10–100 microorganisms are enough to produce disease [17] – it is plausible that recently ‘recovered’ infections were not identified in the faecal samples provided. Prompt testing of food handlers is required to support epidemiological investigations of foodborne outbreaks [11].

Analysis of interactions between food items identified cabbage as an interacting vehicle for infection. Where customers ate both coleslaw and cabbage, the odds of illness were much greater than eating either item on their own, though the effect of cabbage alone was much smaller than that of eating coleslaw without cabbage. Different cabbage types were used for the coleslaw and general salad cabbage in Takeaway A. This may indicate potential cross-contamination, which could have occurred at any number of points in food preparation, storage, or service. However, as it was not possible to collect environmental or food samples from the takeaway, this cannot be confirmed. The EHOs’ inspection identified remediable issues around hygiene and cross-contamination risks. It is possible these risks were present at the time, resulting in contamination of the food and exposure of the cases. Poor hygiene, in particular lack of suitable handwashing facilities in food preparation areas, has been implicated in previous *S. flexneri* outbreaks with foodborne transmission [18].

Thirdly, no potential vehicles outside Takeaway A were associated with illness. This would suggest that the outbreak was not associated with an item in the wider food supply chain and was most likely associated with contamination that occurred on the food premises through potential breaches in hand hygiene and cross-contamination.

Cases in this outbreak were characterized by particularly severe symptoms. Reports of bloody diarrhoea amongst cases in the analytical study were substantially higher than expected (75% reported vs. 10–50% expected [2]). Likewise, almost a third of cases included were hospitalized, substantially higher than the expected admissions rate of 3% [2]. Whilst virulence factors of the isolated organism were not investigated in this instance, it does show the potentially high burden of outbreaks of this nature on local health services, both primary and secondary care.

A strength of this investigation was the use of data from an online app as a source of case and control recruitment for an analytical study. Apps and social networking websites have previously been used by PHW for targeted health promotion to raise awareness of an outbreak [19] but have not been used by the organization to identify and recruit persons when investigating outbreaks linked to the purchase and consumption of food. This novel method reflects changes in the way consumers’ access goods and services, including takeaways. Expedited by the COVID-19 pandemic, ordering food via an app has an estimated annual growth of 11.5% globally [20]. As such, it is likely that this method of case and control recruitment will be employed more frequently in future outbreaks involving food establishments. However, it is essential that the recruitment process using app data is streamlined. By the time these data were made available to the OCT, more than a month had elapsed since the outbreak occurred. As all exposures and key dates (with the exception of microbiological tests) were self-reported in this investigation, it is possible that the delay between exposure and completing the study questionnaire may have introduced recall bias.

Whilst the tests of association used in this investigation were appropriate, the sample size was relatively small. As such, the associations calculated here may not reflect the true effect of the exposure–outcome relationship and should be interpreted with caution.

Due to the lag between exposure and signal detection through microbiological samples, and the pre-planned closure of Takeaway A, it was not possible to obtain relevant food samples for microbiological testing. Therefore, it is not possible to confirm the presence of *S. flexneri* in the coleslaw from Takeaway A. In addition, it was not possible to confirm a human source of infection in any of the staff or their close contacts. We recognize it is possible for initial contamination of cabbage to have occurred in the food supply chain, with practices at the Takeaway increasing the risk of cross-contamination and the notification of a focal outbreak. This would also provide an alternative hypothesis for the lack of genomically related *S. flexneri* isolates identified outside the outbreak cluster. Similar observations were made following a multi-location outbreak of *S. sonnei* linked to contaminated food [21]. However, the associations identified in this analysis, combined with the findings from the environmental investigations of hygiene issues and substandard food preparation practices in the takeaway, would suggest that the source identified in this investigation is plausible.

There is no known biological reason why women would be more likely to become cases. It is possible that the relationship in this case–control study is impacted by the inclusion criteria. In order to be included in the analytical study, cases needed to have a PCR sample positive for *Shigella* sp., or a culture-positive result for *S. flexneri.* Generally, cases will need to access healthcare services (either a GP or hospital) in order to get a specimen pot for microbiological confirmation. A 2013 UK-based study reported that men are 8% less likely to consult a GP than females [22]. As such, the relationship observed in this outbreak may be a reflection of health-seeking behaviours rather than a true association between gender and odds of infection.

## Conclusion

The findings of the case–control study support the hypothesis that consumption of coleslaw from Takeaway A between 10 and 11 February 2023 was associated with this outbreak of shigellosis. Those who ate cabbage as well as coleslaw had a greater odds of illness. Whilst this investigation identified the most likely vehicle of infection, the original source remains undetected. Although uncommon in the UK, *S. flexneri* should be considered as a cause of foodborne outbreaks with a potentially high burden on primary and secondary healthcare services.

Following this investigation, we recommend that environmental health and food standards regulators continue to stress the importance of hand hygiene in catering industries, as a simple, cost-effective measure to curb infection transmission. In addition, health protection authorities should develop guidance to support the ascertainment and use of app data for outbreak investigation purposes.

## Supporting information

Plimmer et al. supplementary material Plimmer et al. supplementary material

## Data Availability

The data used in this investigation contain personally identifiable information. Anonymized information, including that contained in the Supplementary Information, required to reproduce these results is available from the corresponding author on reasonable request.

## Supplementary material

For supplementary material accompanying this paper visit http://doi.org/10.1017/S0950268824000943. click here to view supplementary material
