# Supplementary figures and images for: Large Shigella flexneri outbreak linked to a takeaway, South Wales: a case–control study
Source: Epidemiol Infect. 2025 Feb 14;153:e25. doi: 10.1017/S0950268824000943 (PMC11869068; doi:10.1017/S0950268824000943)

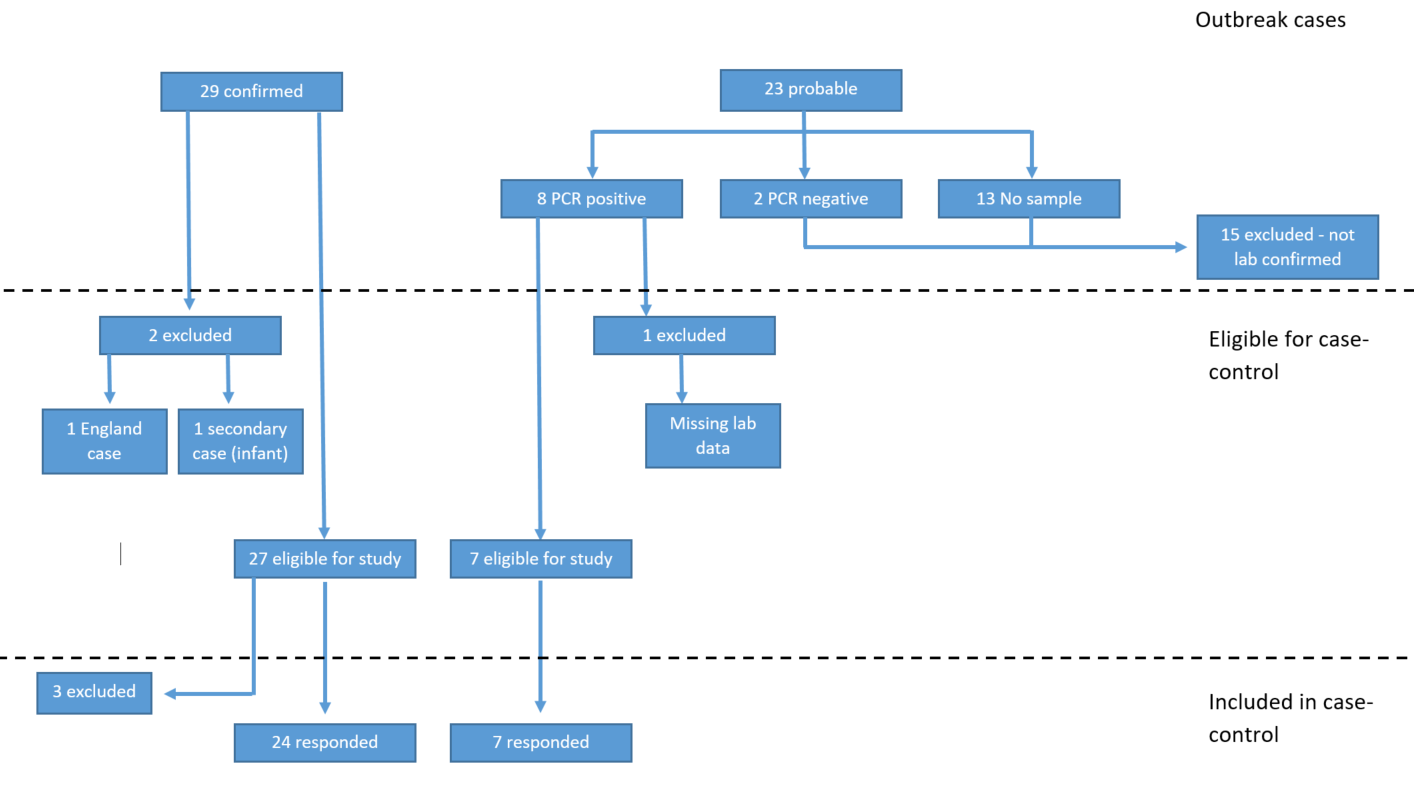

Supplement: Plimmer et al. supplementary material [file S0950268824000943sup001.zip › S.flex case-control_Supplementary_Fig1a.png]

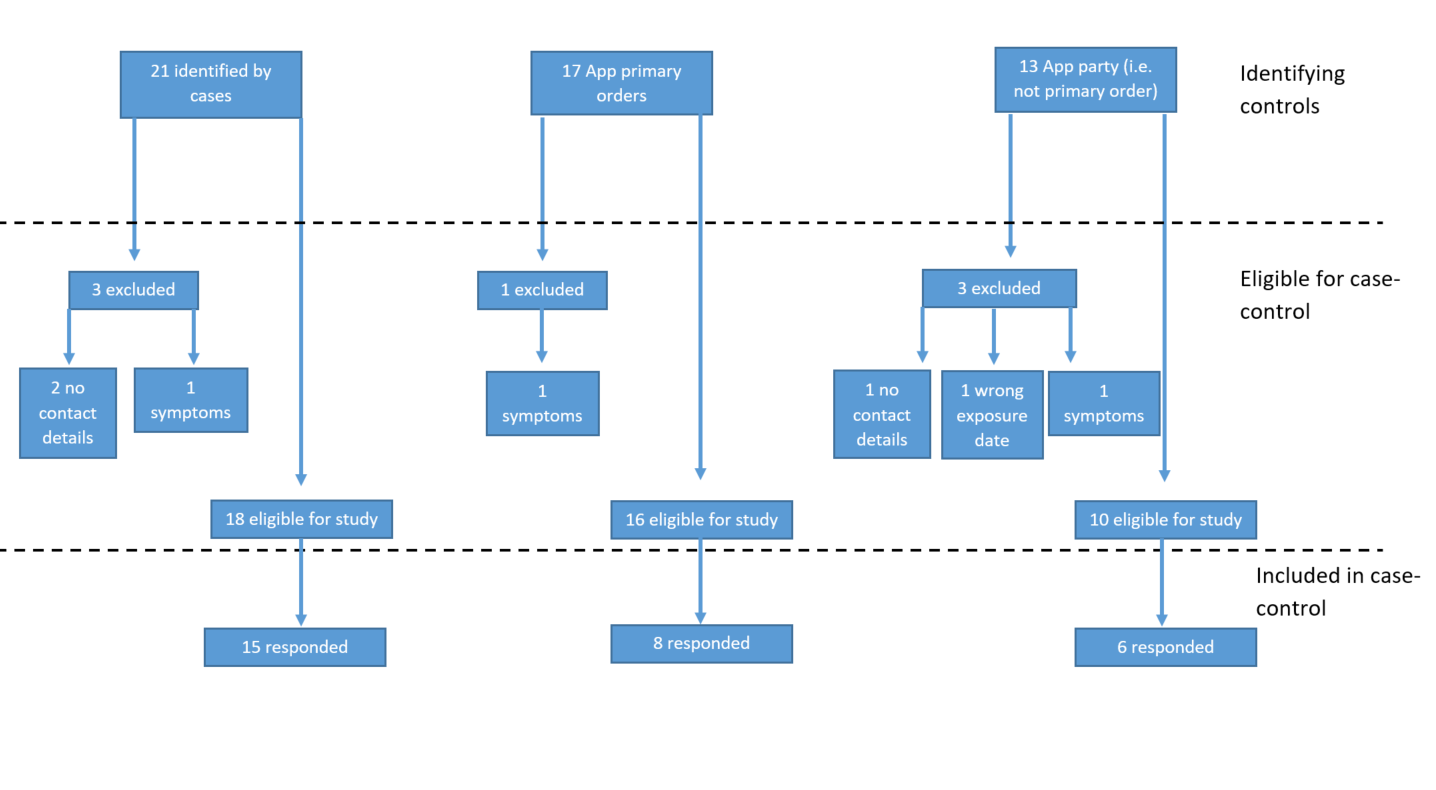

Supplement: Plimmer et al. supplementary material [file S0950268824000943sup001.zip › S.flex case-control_Supplementary_Fig1b.png]

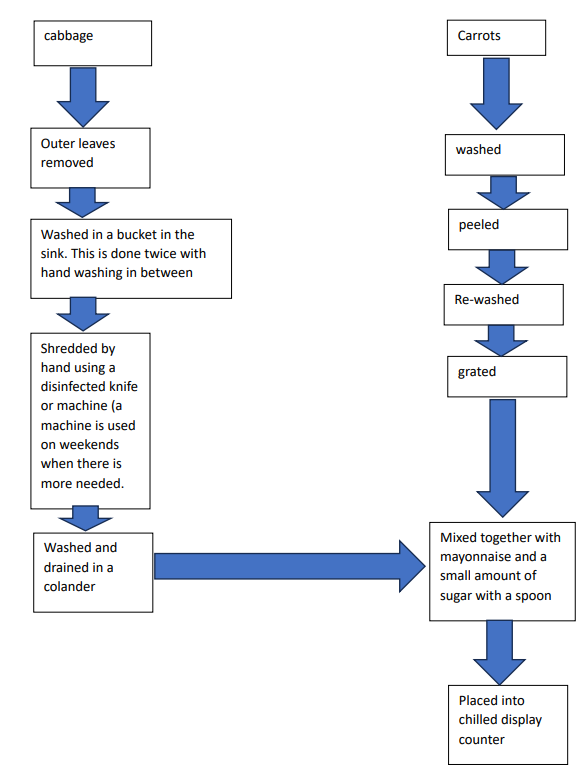

Supplement: Plimmer et al. supplementary material [file S0950268824000943sup001.zip › S.flex case-control_Supplementary_Fig2.png]
